# Whole Genome-Based Characterization of *Listeria monocytogenes* Isolates Recovered From the Food Chain in South Africa

**DOI:** 10.3389/fmicb.2021.669287

**Published:** 2021-07-02

**Authors:** Thendo Mafuna, Itumeleng Matle, Kudakwashe Magwedere, Rian E. Pierneef, Oleg N. Reva

**Affiliations:** ^1^Agricultural Research Council, Biotechnology Platform, Private Bag X05, Onderstepoort, South Africa; ^2^Department of Biochemistry, Genetics and Microbiology, Centre for Bioinformatics and Computational Biology, University of Pretoria, Pretoria, South Africa; ^3^Bacteriology Division, Agricultural Research Council: Onderstepoort Veterinary Research, Pretoria, South Africa; ^4^Directorate of Veterinary Public Health, Department of Agriculture, Forestry and Fisheries, Private Bag X138, Pretoria, South Africa

**Keywords:** cgSNP, cgMLST, AMR, virulence profiles, Benzalkonium chloride resistance, stress tolerance, plasmids, prophages

## Abstract

*Listeria monocytogenes* is an important foodborne pathogen which has the ability to adapt and survive in food and food processing facilities where it can persist for years. In this study, a total of 143 *L. monocytogenes* isolates in South Africa (SA) were characterized for their strain’s genetic relatedness, virulence profiles, stress tolerance and resistance genes associated with *L. monocytogenes*. The Core Genome Multilocus Sequence Typing (cgMLST) analysis revealed that the most frequent serogroups were IVb and IIa; Sequence Types (ST) were ST204, ST2, and ST1; and Clonal Complexes (CC) were CC204, CC1, and CC2. Examination of genes involved in adaptation and survival of *L. monocytogenes* in SA showed that ST1, ST2, ST121, ST204, and ST321 are well adapted in food processing environments due to the significant over-representation of Benzalkonium chloride (BC) resistance genes (*bcrABC* cassette, *ermC, mdrL* and *Ide*), stress tolerance genes (SSI-1 and SSI-2), Prophage (φ) profiles (LP_101, vB LmoS 188, vB_LmoS_293, and B054 phage), plasmids profiles (N1-011A, J1776, and pLM5578) and biofilm formation associated genes. Furthermore, the *L. monocytogenes* strains that showed hyper-virulent potential were ST1, ST2 and ST204, and hypo-virulent were ST121 and ST321 because of the presence and absence of major virulence factors such as LIPI-1, LIPI-3, LIPI-4 and the internalin gene family members including *inlABCEFJ*. The information provided in this study revealed that hyper-virulent strains ST1, ST2, and ST204 could present a major public health risk due to their association with meat products and food processing environments in SA.

## Introduction

*Listeria monocytogenes* remains a considerable public health concern due to its complex ecology and ability to survive in various harsh environmental conditions posed in the food processing facilitates (Ferreira et al., 2014; Hurley et al., 2019; Chen et al., 2020). Assessing the genetic diversity of *L. monocytogenes* is critical in understanding the epidemiology, ecology, and pathogenicity of this pathogen. *Listeria monocytogenes* consists of three major evolutionary lineages including lineages I, II, and III, as well as a rare lineage IV (Chen et al., 2020). These lineages represent 13 recognized serotypes of *L. monocytogenes* which are further grouped into four PCR-serogroups: IIa (1/2a and 3a), IIc (1/2c and 3c), IIb (1/2b and 3b), and IVb (4b, 4d, and 4e) (Doumith et al., 2004; Chen et al., 2020). Molecular typing of *L. monocytogenes* strains can also be done using Multilocus Sequence Typing (MLST), which is based on the sequence variants of seven housekeeping genes to determine their ST and CC. Recently, the cgMLST typing method that takes into account the sequence variation of 1,748 *L. monocytogenes* core genes, has been used to improve isolates discrimination and allowing a standardized comparison with isolate databases for outbreak investigations and surveillance of listeriosis (Moura et al., 2016, 2017).

The adaptation and survival of *L. monocytogenes* in the food processing facilities occur mainly through their ability to proliferate in low temperature, pH and osmotic stress (Takahashi et al., 2014), as well as resistance to sanitation agents and formation of biofilm (Hurley et al., 2019). The control of *L. monocytogenes* in the food processing facilities is mostly based on application of quaternary ammonium compounds (QACs) biocides, such as BCs (Zacharski et al., 2018; Maury et al., 2019). However, the evolution of *L. monocytogenes* resistant to the BCs has been reported in several studies and has become a serious global concern (Zacharski et al., 2018; Korsak et al., 2019). These BC resistances are associated with several efflux resistance genes including *bcrABC* cassette, *Ide, mdrL, qacH, qacA*, *qacE*Δ*1-sul*, and *emrE* which have been reported in various serotypes, ST and CC of *L. monocytogenes* isolated from diverse sources (Kovacevic et al., 2016; Korsak et al., 2019). Furthermore, another key adaptation of *L. monocytogenes* in the environment is the ability to tolerate toxic metals such as arsenic and cadmium (Jesse et al., 2014; Nunes et al., 2016). As result, the co-occurrence of toxic metals and biocide resistance genes in *L. monocytogenes* contribute to the selection of different resistance genotypes and phenotypes that can cause human listeriosis (Angelo et al., 2017; Parsons et al., 2018).

However, despite antibiotic treatment including β-lactam antibiotic such as amoxicillin, penicillin, or ampicillin, and aminoglycosides, such as gentamycin, listeriosis is responsible for mortality rate of 20–30% world-wide (Wang et al., 2015; Wilson et al., 2018). There are reports on *L. monocytogenes* isolates resistant to one or more antibiotics primarily cephalosporins, oxacillin and fosfomycin, particularly in Southern and Western regions of Asia (Sugiri et al., 2014; Wang et al., 2015). The genetic basis of antibiotic resistance in *L. monocytogenes* is associated with different genes such as genes encoding for efflux pumps, particularly for the major facilitator superfamily (*Ide*); erythromycin ribosome methylase (erm) genes (*ermA, ermB*, and *ermC*); tetracycline resistance genes (*tetA, tetK*, and *tetL*)*; fosX*, and *lmrB* (Wilson et al., 2018). The role of mutations in DNA gyrase topoisomerase II (*gyrA* and *gyrB*), topoisomerase IV (*parC* and *parE*) in the development of antibiotic resistance by *L. monocytogenes* was also pointed out by Moreno et al. (2014) and Wilson et al. (2018). The virulence potential of this bacteria is mainly contributed by virulence genes such as *prfA, plcA, hly, mpl, actA, plcB, inlA, inlB*, and *lspA* (Chen et al., 2019).

Several studies in SA have reported the presence of *L. monocytogenes* in food products (Matle et al., 2019; Smith et al., 2019; Thomas et al., 2020). Matle et al. (2019, 2020) conducted a national survey to determine the occurrence and population structure of *L. monocytogenes* strains in meat and meat products isolated from retail, meat processing facilities and abattoirs in SA. Although, this study provides crucial information on meat contamination with *L. monocytogenes*, further investigations are still required to determine the hyper-virulent strains, antibacterial resistance genes, stress tolerance capabilities of *L. monocytogenes* in SA food products. Thus, the objectives of this study were to: (1) use core genome-SNP analysis to determine the genetic relatedness of the most common *L. monocytogenes* strains in SA; (2) assess the genetic basis of the resistance, stress tolerance, genomic localization of the resistance genes in *L. monocytogenes* isolated from food products in SA; and (3) identify key genomic features contributing to virulence potential of *L. monocytogenes* strains in the host.

## Materials and Methods

### Isolates Selection, Genome Assembly, and Annotation

A subset of 152 isolates were selected from a total of 217 isolates from our previous study (Matle et al., 2020). The isolates were selected based on quality of the raw reads and *de novo* assembly in order to avoid false prediction of genes of interest in the present study. Briefly, the raw read quality was assessed with FastQC v.0.11.9 (Andrews, 2010) and the adapters and low-quality reads were trimmed using Trimmomatic v.0.39 (Bolger et al., 2014). SPAdes v.3.13.1 program (Bankevich et al., 2012) was used to create *de novo* assembly of each isolate. The resulting genome assembly were further quality assessed with QUAST v.5.0.2 (Gurevich et al., 2013) and annotated using Prokka v.1.13.7 (Seemann, 2014).

About nine isolates showed poor *de novo* assembly statistics and they were only included in the MLST analysis and subsequently removed from further statistical analysis (Supplementary Table 1). The large scale MLST analysis of *L. monocytogenes* isolates including the isolates of the present study were published by Matle et al. (2020). The cgMLST analysis was also performed using chewBBACA v.3.0 (Silva et al., 2018) only on the isolates used in the present study (a subset of 217 isolates). The cgMLST typing was run with an external schema adapted from BIGSdb-*Lm* platformhttps://bigsdb.pasteur.fr/listeria^1^ (Jolley and Maiden, 2010; Moura et al., 2016). The allele calling on the target genomes were performed with chewBBACA Allele Calling algorithm using the *Listeria_monocytogenes*.trn training file based on the reference strain *L. monocytogenes* EGD-e (acc. No. NC003210). The cgMLST results of these isolates were included as Supplementary Figures 1, 2.

### Core Genome Single-Nucleotide Polymorphism

A reference-based variant calling analysis was performed using the Snippy v.2.6^2^. The annotated genomes were mapped against the complete reference genome of *L. monocytogenes* EGD-e (acc. No. NC003210) with the Burrows-Wheeler Aligner (BWA) v.0.7.12 using default settings (Li and Durbin, 2009). After mapping, the average depths were determined with SAMtools v.1.3 (Li et al., 2009). The variants were called using Freebayes v.0.9.20 (Garrison and Marth, 2012) with the following parameters: minimum base quality of 20, minimum read coverage of 10X, and 90% read concordance at a locus for a variant to be reported. A calling of core genome single nucleotide polymorphisms (SNPs) was produced in Snippy v2.5 to infer a high-resolution phylogeny using Fasttree v.2.1.10 (Price et al., 2010). The total number of SNPs from both inside and outside recombination events were determined with Gubbins (Croucher et al., 2014) using the core alignment file produced by Snippy v2.5.

### Prediction of Virulence Factors, Antimicrobial Resistance, and Stress-Related Genes

Genome assemblies were screened for the presence/absence of genes rendering resistance to antimicrobials, biocides, and heavy metals; and also stress tolerance genes and virulence factors as well as biofilm formation associated genes. ABRicate v0.8.10 was used for this screening with the minimum identity and coverage cut-offs values set by default settings. All alleles for stress tolerance, virulence factors and resistance genes were retrieved from the *Listeria* database hosted by the Pasteur Institute, Paris, France^1^. The biofilm formation associated genes were also retrieved from NCBI (Supplementary Table 3). Other databases used for analyses of virulence factors and resistance genes with ABRicate v0.8.10 were CARD v2.0.3 (Jia et al., 2017), BacMet database (Pal et al., 2014) and Virulence Factor database (VFDB) (Chen et al., 2016). Virulence factors and resistance genes identified by ABRicate v0.8.10 were validated by blastn v.2.10.0^+^.

### Plasmid Reconstruction

Plasmids of the *L. monocytogenes* strains were *de novo* predicted using MOB-suite software (Robertson and Nash, 2018). The MOB-recon algorithm was used to identify plasmid contigs from the draft genomic assemblies. The BLAST-based MOB-recon tool uses markers from sequence databases of known replicons and relaxases in conjunction with a reference database of clustered plasmids provided by MOB-suit software. Finally, the PLSDB web-resource (Galata et al., 2019), a comprehensive large-scale database comprising 13,789 (November 2018) complete sequences of bacterial plasmid, was used for a large-scale comparative analysis to retrieve plasmid records similar to the herein assembled plasmids. The PLSDB database was interrogated using ABRicate v0.8.10^3^ with minimum identity and coverage cut-offs values set by default settings.

### Prediction of Prophages

In order to identify putative prophages, genome assemblies were searched by the PHASTER (PHAge Search Tool–Enhanced Release) server (Arndt et al., 2016). This application scores prophage regions as “intact,” “questionable,” or “incomplete” based on several criteria such as the number of CDSs homologous to certain phages and the percentages of CDSs that match a certain phage. Intact and questionable regions with sequence lengths over 20 kbp were used for the prophage profiling.

### Statistical Validation

Statistical validation of the results was performed using R v.3.6.0^4^ Distribution and association testing were done using Chi-Square tests and over-representation was indicated by a Pearson residual value larger than 2. Additional analysis was done using in-house python scripts.

## Results

### The Core-SNP Phylogenetic Clustering of the Most Common *L. monocytogenes* STs in SA

**FIGURE 1:**
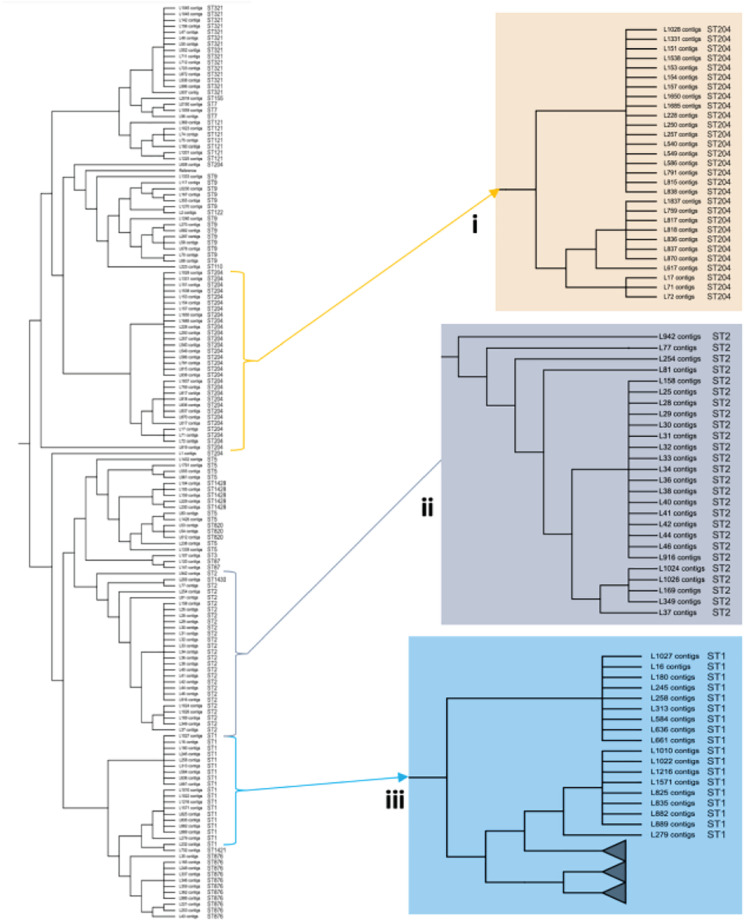
Core-SNP phylogeny showing genetic relatedness of the *L. monocytogenes* strains in SA. **(i)** A section pruned from the original tree showing the South African genetically related ST204 strains. **(ii)** A section pruned from the original tree showing the South African genetically related ST2 strains. **(iii)** A section pruned from the original tree showing the South African genetically related ST1 strains.

**FIGURE 2:**
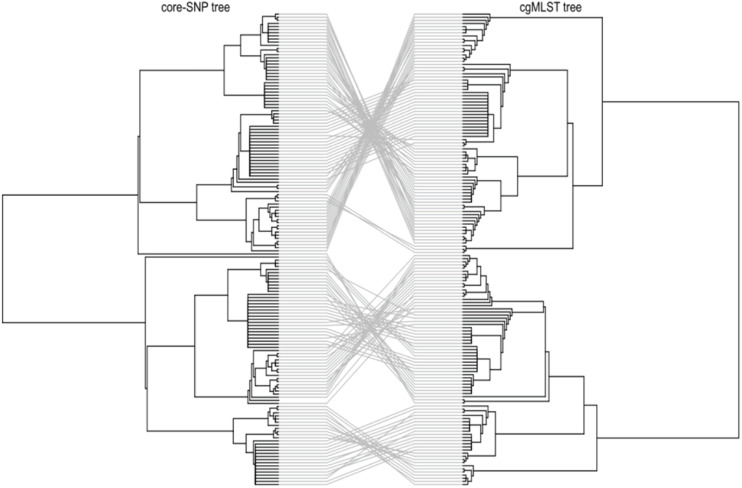
Tanglegram comparison between core-genome SNP (Left) and cgMLST (Right) linking tips with the same label to each of the 152 *L. monocytogenes* isolates.

To investigate the genetic relatedness of the most common *L. monocytogenes* strains in SA, the isolates were mapped against the *L. monocytogenes* EGD-e reference genome and aligned, generating an alignment with core SNPS and a phylogenetic tree. The core-SNP analysis showed that the most frequent ST204 was grouped in three distinct clusters with SNP difference ranging up to 41 SNPs in the core parts of the genomes of these strains (Figure 1). Moreover, the ST1 and ST2 were grouped in two distinct clusters with SNP difference ranging up to 27 and 34 SNPs, respectively (Figure 1 and Supplementary Table 2). These results indicate that SA *L. monocytogenes* isolates belonging to ST1, ST2, and ST204 were generally paraphyletic mixes of diverse genetic variants. Contrary, the strains belonging to ST321 were highly monophyletic and showed maximum two SNPs core genome difference between these isolates (Figure 1 and Supplementary Table 2). Another observation from these results was that ST clustering did not follow the specific isolation sources. In general, the core-SNP phylogenetic tree displayed a good congruence to the cgMLST phylogenetic tree as it is demonstrated in Figure 2. Discrepancies between trees in many cases could be resolved by reordering of the clusters without influencing topologies of the trees.

### Antimicrobial Resistance and Biofilm Formation Genes

**FIGURE 3:**
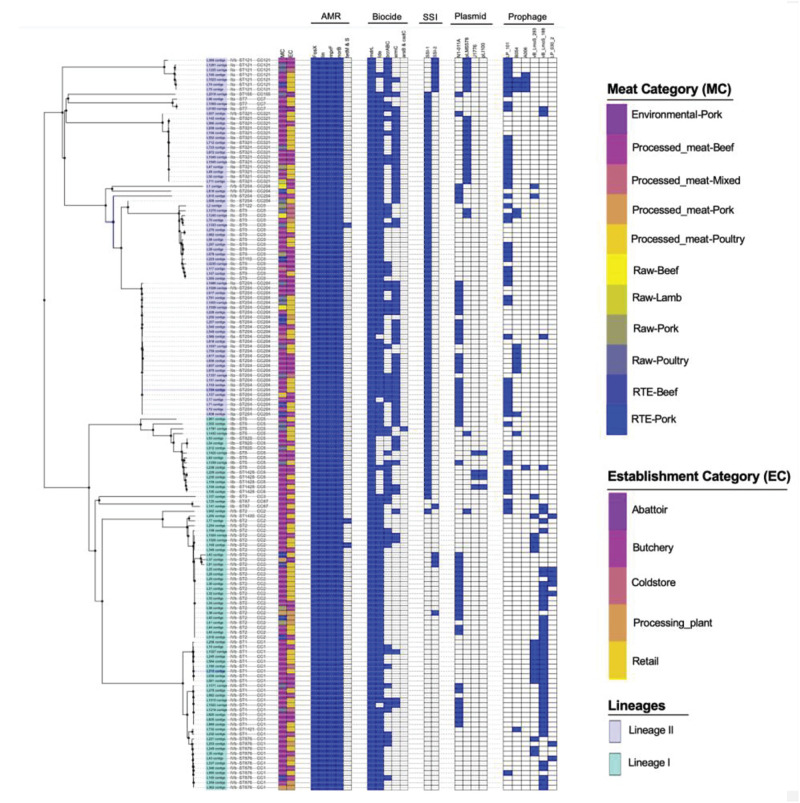
Core-genome MLST phylogenetic grouping of AMR, stress tolerance, biocide resistance genes, plasmids and prophages across the 152 *L. monocytogenes* isolates. The heat map shows the presence (blue) or absence (white) of genes involved in *L. monocytogenes* resistance and virulence. The isolation source MC and EC labeling on the heat map indicate the Meat category and Establishment category, respectively.

The antimicrobial resistance genes were identified in all the isolates of *L. monocytogenes*. These genes include *fosX*, *lin*, *norB*, and *mprF* which confer resistance, respectively, to fosfomycin, lincosamides, quinolones and cationic peptides that disrupt the cell membrane such as defensins (Figure 3). Genes *tetM* and *tetS* that confer resistance to tetracycline were infrequent among isolates. The *tetM* was found only in ST2 and ST9 belonging to serogroups IIb and IVb of lineage I. The *tetS* was observed only in one isolate belonging to ST2 from serogroup IVb of lineage II. Tetracycline resistance genes *tetM* and *tetS* were detected in isolates originated from beef and poultry meat samples obtained from retail and butchery (Figure 3).

Biofilm formation associated genes including *inlL, prfA, actA, lmo0673, bapL, recO, lmo2504*, and *luxS* which play a significant role in survival and persistence of *L. monocytogenes* were analyzed and detected in (*n* = 72, 47%; *n* = 149, 98%; *n* = 72, 47%, *n* = 78, 51%; *n* = 6, 3.9%; *n* = 82, 53%; *n* = 130, 86%; and *n* = 145, 95%) of the isolates, respectively (Supplementary Table 3). The *L. monocytogenes* strains which harbored majority of these genes except for *lmo0673* and *bapL* genes were ST204 and ST321 both belonging to serogroup lla. The well-known ST1 and ST2 isolates which are associated with clinical human listeriosis appear to have less overall biofilm formation associated genes and were also missing the *actA* gene, an important biofilm formation gene. More than 90% of these isolates harbored *prfA, lmo2504*, and *luxS genes.* However, none of the isolates harbored all 8 genes associated with biofilm formation (Supplementary Table 3). Interestingly, *bapL* gene was only specific for ST121 which also harbored most of these genes, but also lacked *actA* gene in the sequenced genomes.

### Benzalkonium Chloride Resistance and Stress Tolerance Genes

The chromosome-borne BC resistance genes including *mdrL* and *Ide*, which are the major facilitator superfamily efflux pumps of *L. monocytogenes* conferring resistance to BC were present in (*n* = 143, 100%) and (*n* = 124, 86.7%) of the isolates, respectively (Figure 3). In many cases, these genes were found in chromosomal inserts of the plasmid-borne BC resistance *bcrABC* cassette (*n* = 55, 38%). The presence of the *bcrABC* cassette was characteristic for ST204 and ST321 all belonging to serogroup IIa of lineage II (*p* < 0.05). Another plasmid-borne BC resistance *ermC* gene was present in (*n* = 58, 40%) of the isolates and was over-represented in ST321 belonging to serogroup IIa of lineage II (*p* < 0.05; Figure 3). No specific over-representation of *Ide*, *bcrABC* cassette and *ermC* was observed in isolates from beef or poultry meat samples (*p* > 0.05). However, the *bcrABC* cassette and *ermC* were significantly over-represented in the isolates from butchery and retail samples (*p* < 0.05; Figure 3).

The stress survival islets (SSI-1 and SSI2), which are known to be responsible for proliferation of *L. monocytogenes* under stressful conditions in food processing facilities, were present in (*n* = 86, 55%) and (*n* = 11, 7.7%) of the isolates, respectively. The SSI-1 was found to be significantly over-represented in ST9, ST204 and ST321 belonging to serogroups IIa and IIc of lineage II (*p* < 0.05; Figure 3). The SSI-2 was found to be significantly over-represented in ST121 belonging to serogroup IIa of lineage II (*p* < 0.05; Figure 3). Islets SSI-2 were over-represented with *p* < 0.05 in the isolates obtained from meat samples from meat processing plants and cold stores in contrast to the distribution of islets SSI-1 showing nor statistically reliable preferences regarding different sources of isolation of *L. monocytogenes* (Figure 3).

### The Assessment of Virulence Factor Genotypes Across Different Serogroups, STs and Isolation Sources

**FIGURE 4:**
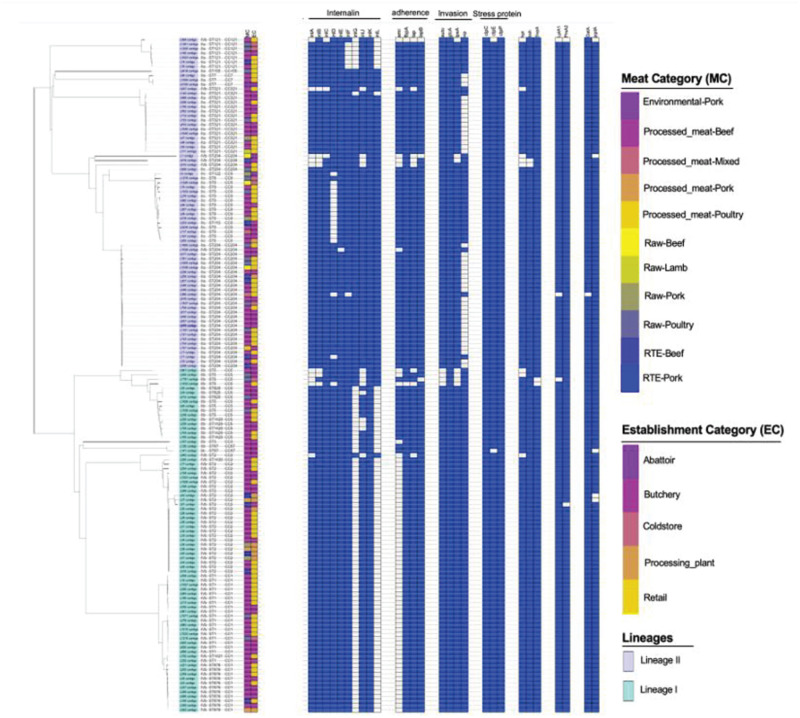
Core-genome MLST phylogenetic grouping of putative virulence factors across the 152 *L. monocytogenes* isolates. The heat map shows the presence (blue) or absence (white) of genes involved in *L. monocytogenes* virulence. The isolation source MC and EC labeling on the heat map indicate the Meat category and Establishment category, respectively.

A total of 68 putative virulence factors were present across the *L. monocytogenes* isolates. The presence and integrity of *Listeria* pathogenicity islands LIPI-1, LIPI-2, LIPI-3, and LIPI-4 were investigated in our previous published study (Matle et al., 2020) and the *Listeria* pathogenicity islands results for the present isolates were included as Supplementary Figure 3. The internalin gene family members including *inlABCEFJK* were present in more than 90% of the isolates. The *inlD* and *inlG* were present in 88 and 47% of the isolates, but absent in ST9 and ST1 which were part of the most abundant ST identified (Figure 4). Other important virulence factors detected in genomes of *L. monocytogenes* isolates include adherence virulence factors such as *ami, fbpA, lap*, and *lapB*, which were present in 54.6, 98.68, 91, and 98% of the isolates; invasion virulence factors *aut*, *gtcA*, *lpeA* and *vip*, which were present in 97, 43, 95, and 72% of the isolates; as well as intracellular survival factors *lplA1*, *prsA2* and *svpA*, which were present in 98, 98.6, and 98.6%, respectively. The *ami*, *gtcA* and *vip* genes were over-represented, respectively in ST204, and ST321; ST1, ST2, ST876; ST1, ST2, ST9, and ST876 (*p* < 0.05; Figure 4).

### The Distribution of *L. monocytogenes* Plasmids Between Different Serogroups, STs and Isolation Sources

A total of four unique plasmids that contribute to the resistance of *L. monocytogenes* to antibiotics were identified in 71% of the tested isolates. Of the four unique plasmids, the most frequent was plasmid N1-011A (*n* = 52, 36.34%), followed by plasmids J1776 (*n* = 28, 19.6%), pLM5578 (*n* = 25, 16.8%), and pLI100 (*n* = 4, 2.6%) across all the study isolates (Figure 3). Plasmid N1-011A was significantly over-represented in ST2 belonging to serogroup IVb of lineage I, and was also over-represented in ST204 belonging to serogroup IIa of lineage II (*p* < 0.05). Plasmid J1776 was over-represented in ST2 belonging to serogroup IVb of lineage I, and was also over-represented in ST9 belonging to serogroup IIc of lineage II (*p* < 0.05). Plasmid pLM5578 was over-represented in ST121 and ST321 belonging to serogroup IIa of lineage II (*p* < 0.05). Plasmid pLI100 was observed only in four isolates belonging to ST1428 of serogroup IIb from lineage I, which also contained plasmid J1776 (Figure 3). The significant association of the plasmids with different isolation sources showed that for the meat category: plasmids N1-011A, J1776, and pLM5578 showed no statistically reliable association with the source of isolation of the pathogen (*p* > 0.05). Contrary, as to the establishment categories: plasmids J1776 and pLM5578 showed a significant association with the retail and butchery category (*p* < 0.05). However, that was not the case with plasmid N1-011A showing no significant associations with either source or establishment categories (*p* > 0.05; Figure 3).

### Prophage (φ) Profiles of *L. monocytogenes* Isolates

Prophage (φ) profiles of the *L. monocytogenes* genomes sequenced in this study were determined using the PHASTER tool for identification and annotation of putative prophage sequences. A total of nine different intact, questionable, or incomplete prophages regions were found across different *L. monocytogenes* isolates (Figure 3). The intact prophage LP_101 [NC_024387] (*n* = 53, 37%) was the most prevalent followed by vB LmoS 188 [NC_028871] (*n* = 45, 31.46%), vB_LmoS_293 [NC_028929] (*n* = 18, 12.58%), and B054 [NC_009813] (*n* = 14, 9%). The LP_101 phage was over-represented in ST121, ST204, and ST321 belonging serogroups IIa of lineage II and also in ST9 belonging to serogroup IIc of lineage II (*p* < 0.05). Phage vB LmoS 188 was over-represented in ST1 and ST2 belonging to serogroup IVb of lineage I (*p* < 0.05). Phage vB_LmoS_293 was over-represented in ST1 belonging to serogroup IVb of lineage I (*p* < 0.05). Phage B054 was over-represented in ST204 belonging to serogroup IIa of lineage II (*p* < 0.05). The significant association of the prophages with different isolation sources showed that for the meat category showed no statistically reliable association with the source of isolation of the pathogen or establishment categories (*p* > 0.05).

## Discussion

The application of the MLST based approach provided important information on the distribution and grouping of genetically related *L. monocytogenes* strains in the SA food processing environment. In the present study, a total of four serogroups represented by 19 STs belonging to 11 different CCs which are a group of closely related STs were identified and classified to lineage I and lineage II using the cgMLST analysis (Supplementary Figures 1, 2; Matle et al., 2020). The current study revealed that the most prevalent serogroups among SA isolates were IVb and IIa, which have also been found to be over-represented in food sources in other countries and were causative agents of more than 80% of global *L. monocytogenes* infections in human (O’Connor et al., 2010; Jamali et al., 2015; Lee et al., 2018). The most prevalent STs were ST204 and ST321 belonging to lineage II, which were mainly found in foods and food processing environments. Other common isolates were ST1 and ST2 belonging to lineage I, which are highly associated with clinical human listeriosis and demonstrate an enhanced pathogenetic potential (Maury et al., 2019; Matle et al., 2020; Palma et al., 2020). The *L. monocytogenes* strains and variants reported in the present study have been shown to be globally distributed and able to survive and persist for months and even years in food-processing environments and to be kept in contaminating food products in food processing environments for long time (Knudsen et al., 2017; Harrand et al., 2020; Matle et al., 2020).

The current study showed a paraphyletic variability of isolates ST1, ST2, and ST204, which differed by up to 41 SNPs in their core genome sequences contrasting them from ST321 isolates, which showed a significant level of conservation of their core genome with not more than two SNPs difference between them. It shows that ST1, ST2, and ST204 variants potentially are more dangerous in generating unusual genetic variants of the pathogen causing disease outbreaks. A study by Li et al. (2017) also reported a significant genetic variability of different *L. monocytogenes* isolates from foods demonstrated by SNP calling. Grouping of isolates by their core-SNP displayed a good congruence with cgMLST clustering; however, it should be noted that the strains grouped into clusters by these two methods still may show quite different pathogenicity potentials due to absent or present of different resistance and virulence genes located within chromosomes, plasmids and prophages (Li et al., 2017; Blanc et al., 2020).

Recent studies on antimicrobial resistance of *L. monocytogenes* have typically reported low levels of antimicrobial resistance in isolates from the food production environments. These reports were based on several studies performed in SA, Europe and Asia (Li et al., 2016; Matle et al., 2019; Wilson et al., 2018). The present study has reported that various antibiotic resistance genes, including *fosX*, *lin*, *mprF*, *norB*, and *mgrA*, were present in all the isolates including the strains from food processing environments. This global trend to a wider distribution of the antibiotic resistant genes in *L. monocytogenes* population was reported in a recent publication by Wilson et al. (2018). The repertoire of resistance genes typical for *L. monocytogenes* is enriching other genes, particularly by tetracycline resistance genes *tetM* and *tetS* found in a few isolates belonging to ST2 and ST9 of serogroups IVb and llb, which were isolated from butchery and retail. These genes have been detected previously in *L. monocytogenes* strains isolated from food and environmental samples (Escolar et al., 2017; Olaimat et al., 2018). Although, tetracycline is believed to be the most frequent resistance trait in *L. monocytogenes* isolated from human and food processing environments, the present study found tetracycline resistance genes only in few *L. monocytogenes* isolates, which most likely were acquired by *L. monocytogenes* with conjugative plasmids and transposons originating from *Enterococcus* or *Streptococcus* as result of horizontal gene transfer (Olaimat et al., 2018).

The key factors of adaptation and survival of *L. monocytogenes* in the food processing environments is the ability to develop resistance to QACs, such as BC, through the activity of efflux pumps encoded by *qacH* and genes of the *brcABC* cassette (Horlbog et al., 2018) and biofilm formation. The present study identified several chromosome-borne BC resistance genes, *mdrl* and *ide*, that confer tolerance to BCs in all the isolates. A study by Conficoni et al. (2016) also reported the presence of *mdrl* and *ide* in isolates from meat-processing environment that agrees with the present study. Several other genes, such as *ermC emrE, qacH*, and *bcrABC* cassette, also are responsible for tolerance to BC, a very common compound of sanitizers which is used in food industry (Kovacevic et al., 2016; Muhterem-Uyar et al., 2018; Kurpas et al., 2020). The present study identified *bcrABC* cassettes in 38% of isolates and the plasmid-borne *ermC* gene in 40% of the isolates belonging to serogroup IIa (ST121, ST204, and ST321) of lineage II, which suggests that these isolates are well adapted to survival in the food-processing environment where QACs are commonly used as sanitizers. Indeed, it was shown in the current study that these genes were over-represented in retail and butchery. Identification of drug resistance genes performed in this study may not be comprehensive due to inability to complete whole genome sequences of the isolates. Particularly, several well-known *Listeria* resistance genes such as *emrE* (Kovacevic et al., 2016) and *qacH* carried with Tn6188 (Horlbog et al., 2018) were not found when the sequences were searched against the BacMet database. Additionally, nucleotide sequences of these genes were obtained from the database of *Listeria* genes hosted at http://bigsdb.pasteur.fr/listeria/ and blasted against the assembled contigs of the *Listeria* isolates. This search didn’t retrieve any significant matches. Either these genes were absent in the sequenced genomes, or they were fragmented in the contigs sequences. The SSI-1, which has been linked to tolerance toward acidic, bile, gastric, and salt stresses, was present in 55% of the isolates and was found to be significantly over-represented in ST9, ST204, and ST321 belonging to IIa serogroup of lineage II (*p* < 0.05). The SSI-2, which is responsible for survival under alkaline and oxidative stresses (Harter et al., 2017), was found to be significantly over-represented in ST121 from lineage II isolated from processing plant and cold store categories (*p* < 0.05). These results corroborate with a previous study (Hurley et al., 2019) showed that SSI-2 was only found in ST121, whereas SSI-1 was distributed in various STs from both lineages I and II. Co-occurrence of BC resistance genes with the stress response genes revealed by the current study implies a serious hygiene management concern. The only available data with regard to the resistance of *L. monocytogenes* to disinfectants applied in food production environments refer to genotypic resistance to QACs. Dilution in the environment and biodegradation result in QAC concentration gradients and as a result, the microorganisms are frequently exposed to sub inhibitory concentrations of QACs. The low-level resistance to QACs in *L. monocytogenes* may contribute to its environmental adaptation and persistence (Martínez-Suárez et al., 2016). Therefore, a need exists to evaluate the use of QACs disinfectants groups and the occurrences of resistance in food production facilities in SA and worldwide. Moreover, the present study also showed that ST204 and ST321 appear to have high ability of biofilm formation capacity which contribute to *L. monocytogenes* adaptation and survival in food processing environment. These results corroborate with a previous study (Pasquali et al., 2018; Stoller et al., 2019) showed that these strains have high biofilm forming capacity under specific environmental conditions. Pasquali et al. (2018) showed that the biofilm formation associated *actA* gene was truncated in all ST121 isolates. Similar trend was observed in the present study were *actA* gene was not detected in all the ST121, ST1, and ST2 isolates. This *actA* gene is known to be responsible for polymerization of actin which is important for motility of *L. monocytogenes* within the host cell as well as in the first steps of biofilm formation (Travier et al., 2013; Pasquali et al., 2018).

The pathogenic potential of a given *L. monocytogenes* strains is determined by the functionality of a large number of genes known as “virulence factors,” all of which have different roles at various stages of the infection cycle. The present study assessed for the presence of 115 putative virulence markers that could be used to predict the level of potential virulence of *L. monocytogenes* isolates. It was suggested to classify isolates of this species as putatively hypo-virulent, with unknown virulence potential, and putatively hyper-virulent (Hurley et al., 2019). A total of 68 virulence markers were identified across the isolates suggesting that most virulence markers are ubiquitous across *L. monocytogenes* strains in SA. Intact LIPI-1, which harbor Prf-A dependent virulence cluster genes that are critical in the infectious cycle of *L. monocytogenes*, was mostly presented in ST1 and ST876 isolates from serogroup IVb belonging to lineage I, and also in ST9, ST204, and ST321 from serogroups IIa and IIc belonging to lineage II (Supplementary Figure 3; Matle et al., 2020). LIPI-1 has been reported to be the first identified pathogenicity island in *L. monocytogenes* distributed across different *L. monocytogenes* strains (Chen et al., 2020). In the present study, the LIPI-3, which is associated with enhancing the virulence capabilities of *L. monocytogenes*, was found ubiquitous in ST1 from serogroup IVb belonging to lineage I, but was also present in 2 isolates from lineage II belonging to ST204 (Supplementary Figure 3; Matle et al., 2020). The LIPI-3 Island carries a gene encoding the hemolytic and cytotoxic factor known as listeriolysin S, which contributes to the intracellular survival of *L. monocytogenes* in human polymorphonuclear neutrophils (Clayton et al., 2011; Hurley et al., 2019). Painset et al. (2019) and Chen et al. (2020) reported similar findings and revealed that LIPI-3 is ubiquitous to lineage I, which was also observed in the present study. Hyper-virulent strains have also been shown to possess the recently described pathogenicity island LIPI-4 that confers hyper-virulence by enhancing the invasion of the CNS and placenta (Grad and Fortune, 2016; Maury et al., 2016). The LIPI-4 Island was identified in the present study in 3.2% of the isolates belonging to serogroup IIb and IVb (ST2 and ST87) from lineage I (Supplementary Figure 3; Matle et al., 2020). While isolates of ST1, ST2, ST204, and ST321 generally were characterized with an abundance of virulence genes. However, the known adhesion and invasion related genes, *aut, inlF, inlJ*, and *vip*, were not found in genomes of these microorganisms which suggests a possible limitation of the invasiveness and virulence of this *L. monocytogenes* strains (Lindén et al., 2008; Martins et al., 2012). The *inlA gene* was found in more than 90% of the isolates in the current study. A recently published work on the same *L. monocytogenes* isolates revealed the truncation of the gene *inlA* due to premature stop codon, which has been associated with reduced invasiveness in some *L. monocytogenes* strains (Matle et al., 2020). This mutation may serve as a marker of hypo-virulence. Analysis of translated *inlA* protein sequence from isolates in this study identified 18 isolates, all from ST121 and ST321 of lineage II having this mutation reported for the first time for SA isolates (Matle et al., 2020).

This study suggested an important role of virulence plasmids of *L. monocytogenes* to confer increased tolerance to multiple stress condition in food processing environments. Blasting of nucleotide sequences of the found plasmids against NCBI database revealed homology of these plasmids at more than 90% similarity with the virulence plasmid N1011A, pLM5578, J1776, and pLI100 common for *L. monocytogenes* isolates (Palma et al., 2020). The majority of plasmids N1011A and pLM5578 isolates also carried *bcrABC* cassette suggesting a high correlation between the presence of these plasmids and BC tolerance in *L. monocytogenes* strains. Plasmid N1011A was associated with the most common isolates of serogroups IVb and IIa (ST204 and ST2), while pLM5578 was associated with serogroup IIa (ST121 and ST321) suggesting an importance of this plasmids in contribution to survival of hyper-virulent *L. monocytogenes* strains in the food processing environments (Kuenne et al., 2010). Furthermore, Kropac et al. (2019) showed that small plasmid pLMST6 which harbor *emrC* gene confers increased BC tolerance in *L. monocytogenes.* Plasmid PLMST6 was not detected in the present study. In addition to the virulence plasmids, nine prophages were distributed across the *L. monocytogenes* isolates from different sources. Analysis of the genetic repertoire of these prophages suggested their possible involvement in virulence and resistance. ST1, ST2, ST204, and ST321 displayed the highest numbers of prophages per genomes. This shows that adaptation of *L. monocytogenes* to specific environmental niches in food processing industry and short-term evolution of both distantly and closely related *L. monocytogenes* strains have been linked to the diversification of these prophages (Harrand et al., 2020; Palma et al., 2020).

## Conclusion

The findings of this study that was based on NGS sequencing of *L. monocytogenes* isolates revealed the overall contribution of plasmids, prophages chromosomal genes toward pathogenicity and adaptation to meat processing and storage environment. The study showed that ST1, ST2, ST121, ST204, and ST321 were the most frequent among isolates and well adapted to survive in food processing environments in SA. Several hyper-virulent strains were revealed among isolates belonging to ST1, ST2, and ST204, which could present a major public health risk due to their association with meat products and food processing environments in SA, whereas hypo-virulent isolates from both lineage I and II belonged to ST121 and ST321. The information provided in this study is important for enhancing our understanding of the adaptation and survival of this pathogen in the food-processing environments. Also, the obtained results will aid in developing new approaches to assess the virulence potential of *L. monocytogenes* isolates and the efficacy of using BC disinfectants in food-processing facilities in SA.

## Data Availability Statement

The datasets generated during and/or analyzed during the current study are available in the NCBI Sequence Read Archive (SRA) repository, accession number: PRJNA720786.

## Ethics Statement

Ethical approval was obtained from University of Pretoria, Faculty of Natural and Agricultural Sciences Research Ethics Committee (NAS324/2020). All methods in this study were approved by University of Pretoria, Faculty of Natural and Agricultural Sciences Research Ethics Committee, and performed in accordance with the relevant guidelines and regulations.

## Author Contributions

RP, IM, KM, and OR: conceptualization. OR and RP: supervision. TM: writing original draft preparation, methodology, bioinformatics, and statistical analysis. RP, OR, and IM: manuscript review and editing. IM and KM: funding acquisition. All authors have read and agreed to the published version of the manuscript.

## Disclaimer

The views presented in this article are those of the authors and do not represent an official position of the authors’ affiliated institutions.

## Conflict of Interest

The authors declare that the research was conducted in the absence of any commercial or financial relationships that could be construed as a potential conflict of interest.

## Footnotes

1 https://bigsdb.pasteur.fr/listeria

2 https://github.com/tseemann/snippy

3 https://github.com/tseemann/ABRicate

4 https://www.R-project.org/
