# Supplementary material for: Whole Genome-Based Characterization of Listeria monocytogenes Isolates Recovered From the Food Chain in South Africa
Source: Front Microbiol. 2021 Jul 2;12:669287. doi: 10.3389/fmicb.2021.669287 (PMC8283694; doi:10.3389/fmicb.2021.669287)
Supplement: Supplementary file 1 [file Data_Sheet_1.PDF]

Supplementary Materials

Whole genome-based characterization of *Listeria monocytogenes* Isolates recovered from the Food Chain in South Africa.

T. Mafuna[1, 3], I Matle[2], K Magwedere[4], R.E. Pierneef[1], O.N Reva[3]

[1]Agricultural Research Council, Biotechnology Platform, Private Bag X05, Onderstepoort, 0110, South Africa

[2]Bacteriology Division, Agricultural Research Council: Onderstepoort Veterinary Research, Onderstepoort, Pretoria, South Africa

[3]Centre for Bioinformatics and Computational Biology; Department of Biochemistry, Genetics and Microbiology; University of Pretoria; South Africa

[4]Directorate of Veterinary Public Health, Department of Agriculture, Forestry and Fisheries, Private Bag X 138, Pretoria, 0001, South Africa

Corresponding author: oleg.reva@up.ac.za, +27 (0)12 420 5810

Keywords: sequence type, PCR serogroups, cgSNP, cgMLST, AMR, virulence profiles, Benzalkonium chloride resistance, stress tolerance, plasmids, prophages

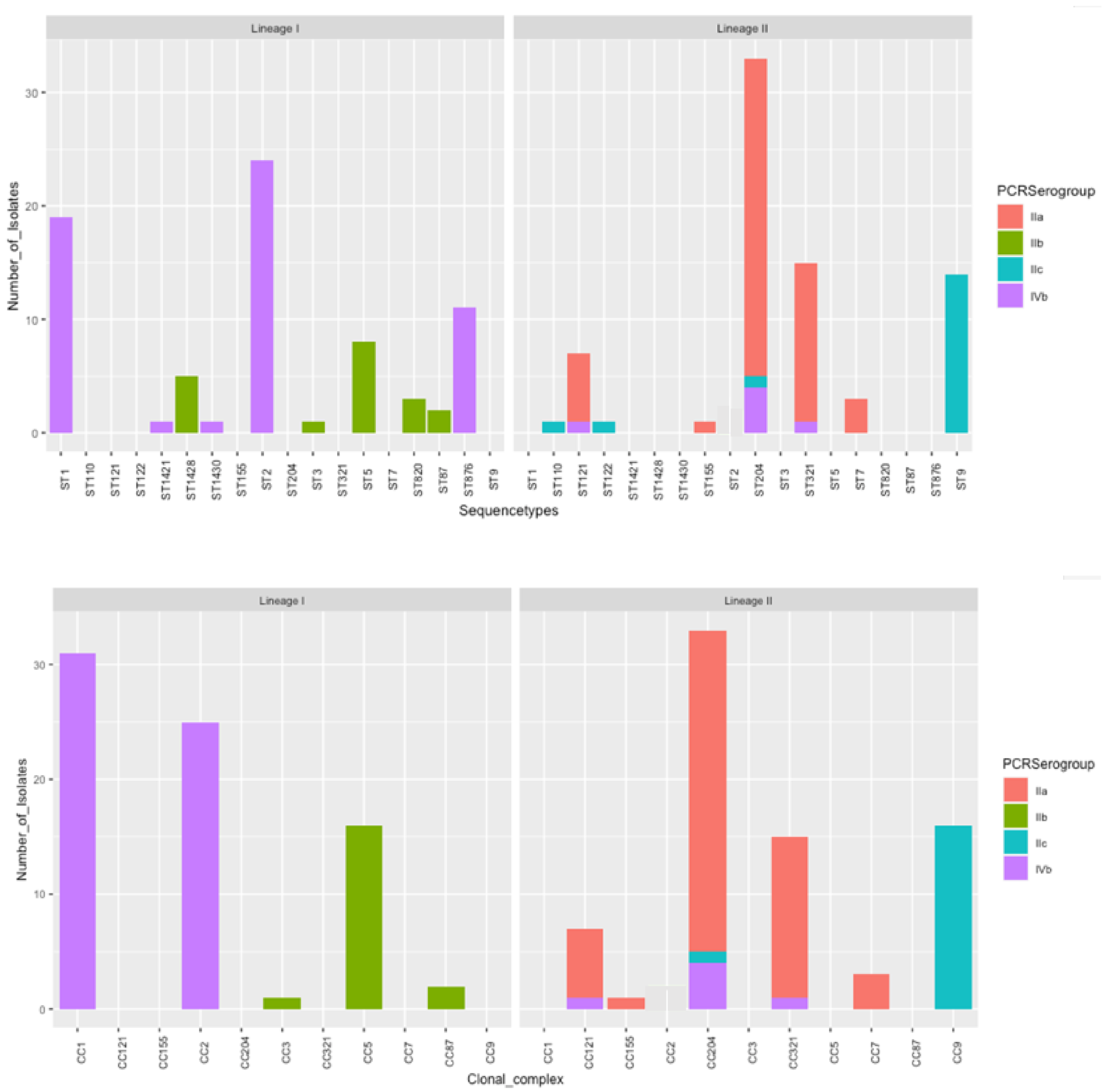

Figure S1. Distribution of *Listeria monocytogenes* serogroups, STs and CCs per study isolates. The coloured bar graph on lineage I indicate serogroups, sequences types and clonal complexes that belong to lineage I. The coloured bar graph on lineage II indicates serogroups, sequence types and clonal complexes that belong to lineage II.

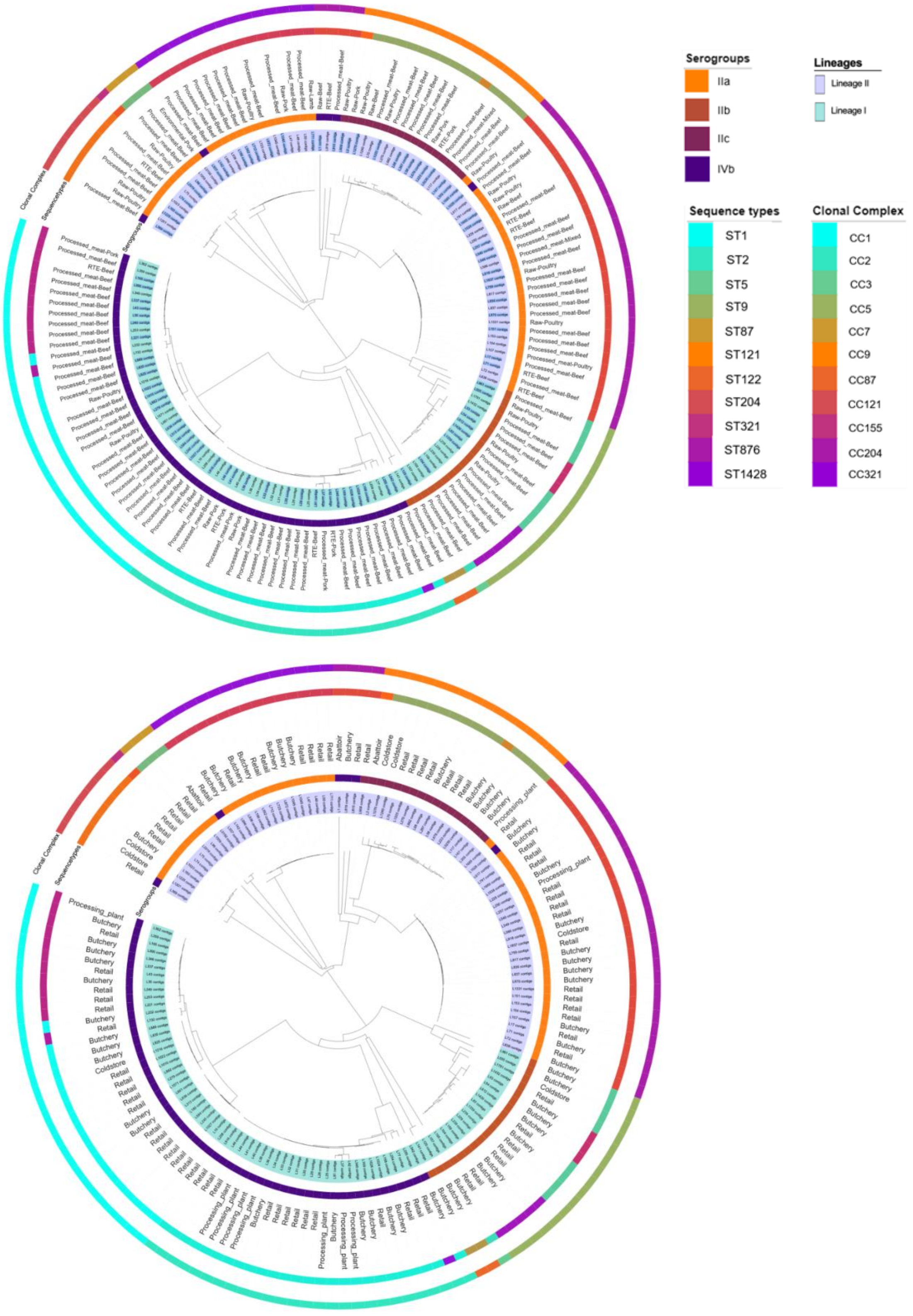

Figure S2. Core genome MLST phylogenetic analysis of *Listeria monocytogenes* isolates showing the association between MLST typing and isolation source isolates, (**A**) Meat category and (**B)** Establishment category isolates.

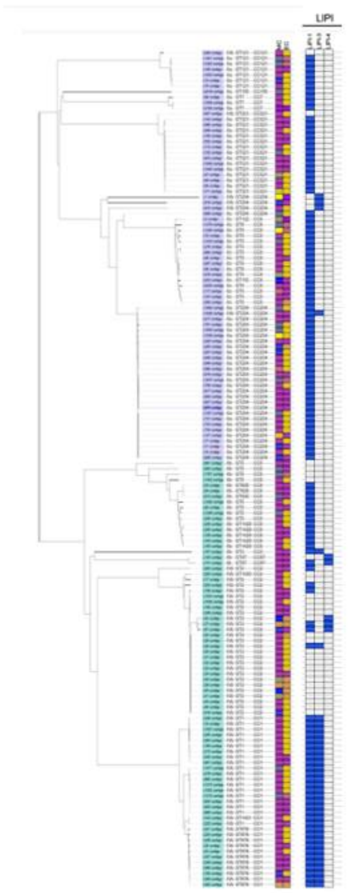

Figure S3. Core-genome MLST phylogenetic grouping of *Listeria* pathogenicity island across the *L. monocytogenes* isolates. The heat map shows the presence (blue) or absence (white) of genes involved in *L. monocytogenes* virulence. The isolation source MC and EC labelling on the heat map indicate the Meat category and Establishment category, respectively.
